# Response to: Association of Intraoperative Red Blood Cell Transfusions With Venous Thromboembolism and Adverse Outcomes After Cardiac Surgery

**DOI:** 10.1097/AS9.0000000000000268

**Published:** 2023-02-20

**Authors:** Jacob Raphael, Xiaoke Feng, Matthew S. Shotwell, Michael A. Mazzeffi, Bruce A. Bollen, Ashish S. Shah, Miklos D. Kertai

**Affiliations:** From the * Department of Anesthesiology, Thomas Jefferson University Hospitals, Philadelphia, PA; † Department of Biostatistics, Vanderbilt University Medical Center, Nashville, TN; ‡ Department of Anesthesiology, Vanderbilt University Medical Center, Nashville, TN; § Department of Anesthesiology and Critical Care Medicine, George Washington University School of Medicine and Health Sciences, Washington DC; ‖ Missoula Anesthesiology PC, Missoula, MT; ¶ Department of Cardiac Surgery, Vanderbilt University Medical Center, Nashville, TN.

We are grateful for the interest and comments of Butt et al^1^ with regard to our recently published study.^2^

First, Butt et al^1^ noted that the overall exposure rate to intraoperative packed red blood cells (PRBCs) was 17.8% in our study compared to the reported rate of 12.8% to 60.3% in previous studies in similar patients. They then speculated that the relatively lower rate of intraoperative PRBC transfusion observed in our study was due to our decision to label missing information on intraoperative PRBC transfusion as “no transfusion.” In support of their argument, they cited a retrospective study of 8141 patients from 10 centers who had cardiac surgery between 2011 and 2014; these patients underwent either isolated coronary artery bypass grafting (CABG) surgery or aortic valve replacement, and Butt et al^1^ did not cite the risk-adjusted rate for intraoperative PRBC transfusion, which was found to be 4% to 59%.^3^ Thus, our reported rate of 17.8% is likely reflective of the more contemporary and average national rate of intraoperative PRBC transfusion for isolated CABG surgery. Furthermore, Butt et al^1^ highlighted that labeling missing data for intraoperative PRBC transfusions as “no transfusion” as justified in the methodology section of our manuscript, and omission of any postoperative transfusion before any hospital-acquired venous thromboembolic (HA-VTE) complications might have biased the association between intraoperative PRBC transfusion and HA-VTE complications. To address this, we performed a sensitivity analysis which indicated that after removing patients with missing data for intraoperative PRBC transfusion the association between intraoperative PRBC transfusion and HA-VTE remained (odds ratios [ORs] for 1, 2, 3, and ≥4 units of PRBC versus none: 1.16 [1.09 to 1.23], 1.19 [1.12 to 1.26], 1.59 [1.49 to 1.68], 1.49 [1.40 to 1.58]). Postoperative transfusion before any HA-VTE complications was not collected by the Society of Thoracic Surgeons Adult Cardiac Surgery Database and was not available for analysis.

Second, Butt et al^1^ also indicated that a causality relationship between intraoperative PRBC transfusion and HA-VTE complications was not supported by the lower odds of HA-VTE complications in women. To address this comment, we reiterate the following: i) given the retrospective nature of our study it was not meant to definitively ascertain a causality, but rather reinforce a previously described association between intraoperative PRBC transfusion and HA-VTE complications in a large-scale national registry; ii) our propensity score-weighted, and covariate-adjusted analysis including adjusting for sex indicated that intraoperative PRBC transfusion was independently associated with HA-VTE complications after isolated CABG surgery; iii) only a small proportion of patients had missing data for height (0.03%) and weight (0.02%), and thus, the calculated body mass index (BMI) was observed in nearly all patients (missing, 0.03%). Therefore, our conditional imputation for 0.03% of the missing BMI is highly unlikely that it may have impacted the association of BMI and intraoperative PRBC transfusion in the development of the propensity score. We agree with Butt et al^1^ that it would have been potentially valuable to study the impact of geographic location on our selected patient- and procedure-related characteristics, but this information was not available through the participant user file research mechanism via which our study was approved.

Third, we agree with the comment that delayed thromboprophylaxis during a prolonged hospital length of stay may also exacerbate HA-VTE complications independently or in combination with intraoperative and postoperative PRBC transfusions. As indicated by Butt and colleagues^1^ the Society of Thoracic Surgeons Adult Cardiac Surgery Database does not capture data on delayed thromboprophylaxis during a prolonged hospital length of stay, and thus we were not able to study its respective association with prolonged length of stay.

Butt et al^1^ speculate that our approach of implementing a propensity score-weighted method using matching inverse probability of treatment weighting to account for potential confounding of the association between intraoperative PRBC transfusion and our outcomes may have been impacted by handling of extreme weights.^4^ To address the possibility of residual confounding, we considered the evidence for causality (*E* value),^5^ which is considered as an alternative approach to sensitivity analyses for potential unmeasured confounding, and were able to demonstrate that there was a low potential for unmeasured confounding significantly influencing the association between intraoperative PRBC transfusion and our primary outcome of HA-VTE. Finally, we agree with Butt et al^1^ that there is a need to better predict HA-VTE. We also strongly believe that there is a potentially unmet need for current and future “studies to explore interventions aimed at improving perioperative anemia and blood management while maintaining appropriate postoperative thromboprophylaxis, thereby significantly decreasing the need for PRBC transfusions in this setting and potentially decreasing the incidence of postoperative venous thromboembolism after cardiac surgery.”^2^
